# Editorial: Community series in: clinical and genetic determinants of diabetes and complications, volume II

**DOI:** 10.3389/fendo.2026.1895212

**Published:** 2026-06-12

**Authors:** Kexin Zhang, Maurizio Delvecchio, Xiaodong Sun

**Affiliations:** 1 Department of Endocrinology and Metabolism, Shandong Provincial Key Medical and Health Laboratory of Endocrinology and Metabolic Diseases, Affiliated Hospital of Shandong Second Medical University, Weifang, China; 2 Department of Biotechnological and Applied Clinical Sciences, University of L’Aquila, Aquila, Italy

**Keywords:** diabetes mellitus, diabetic complications, environment, genetics, risk factors

Diabetes mellitus remains one of the most pressing global health challenges, not only because of its increasing prevalence but also because of its extensive burden of acute and chronic complications (1–3). Although hyperglycemia is the defining feature of diabetes, its clinical consequences extend far beyond glucose metabolism, involving the retina, kidney, cardiovascular system, liver, nervous system, reproductive health, and immune-metabolic pathways (3, 4). The heterogeneity of diabetes across disease type, age, sex, body composition, reproductive history, and comorbid conditions makes uniform approaches to screening and management insufficient (5–7). Accordingly, contemporary diabetes research is increasingly moving toward earlier risk prediction, biomarker-driven stratification, organ-specific assessment, and individualized intervention. This Research Topic compiles 16 studies that highlight this evolving landscape by integrating evidence from cohort studies, prediction models, systematic reviews, meta-analysis, case reporting, and bibliometric mapping.

A central message emerging from this collection is the shift from reactive treatment to proactive risk identification. Several studies demonstrate that routinely available clinical indicators can be reorganized into practical tools for early detection and prevention. Gao et al. showed that the total cholesterol–high-density lipoprotein–glucose index was independently associated with incident diabetes, supporting the value of combined lipid–glucose indices for identifying metabolically vulnerable individuals before overt disease develops. Extending this preventive concept to diabetic complications, Yang et al. developed and externally validated a simple nomogram based on diabetes duration, HbA1c, and BMI for diabetic retinopathy risk prediction in primary care. Zhang et al. further applied this early-warning approach to acute metabolic complications, showing that a lower hemoglobin-to-red blood cell distribution width ratio was independently associated with recurrent diabetic ketoacidosis in patients with type 2 diabetes. Together, these findings suggest that effective risk prediction does not always require complex or costly technologies; accessible clinical variables can provide meaningful signals for closer monitoring and timely intervention.

Diabetic kidney disease (DKD) represents a particularly important focus of this Research Topic and illustrates how diabetes research is moving beyond conventional markers. Traditional DKD assessment relies heavily on albuminuria and eGFR, but these measures may miss early tubular dysfunction, non-albuminuric injury, or molecular vulnerability before irreversible renal damage occurs. Zhang et al. addressed this gap by developing and externally validating the FluxPro-DKD fusion model, which integrates digital urine physicalomics with paired serum–urine metabolomics flux ratios to identify early DKD and urine albumin-to-creatinine ratio (UACR)-silent risk. Hao et al. further advanced biomarker discovery using UK Biobank proteomic and metabolomic data, identifying plasma growth differentiation factor 15 as a strong predictor of future diabetic kidney complications that outperformed blood glucose and HbA1c for 5-year DKD prediction. Complementing these multi-omics approaches, Wang et al. showed that plasma R-Spondin 2 levels increased with worsening Kidney Disease: Improving Global Outcomes (KDIGO) risk category and were correlated with UACR, urea, serum creatinine, and estimated glomerular filtration rate. Collectively, these studies indicate a conceptual transition in DKD research from detecting established renal dysfunction to identifying earlier molecular, tubular, and systemic signatures of kidney vulnerability, potentially allowing intervention before irreversible renal injury develops.

The studies on organ-specific complications further show that diabetes-related damage should be understood as interconnected rather than isolated. Lu et al. compared anti- vascular endothelial growth factor agents combined with pars plana vitrectomy for proliferative diabetic retinopathy and showed that different agents offer distinct perioperative advantages, emphasizing individualized therapeutic selection according to surgical goals and patient priorities. Moving from ocular microvascular disease to cardiovascular risk, Kong et al. found that elevated fasting glucagon was independently associated with coronary artery disease in women with type 2 diabetes, suggesting that sex-specific metabolic markers may refine cardiovascular risk assessment. García-Sáenz et al. expanded the complication spectrum to liver disease in adults with type 1 diabetes by evaluating non-invasive approaches for metabolic dysfunction-associated steatotic liver disease and fibrosis risk stratification. Beyond vascular and hepatic complications, neurological outcomes are also gaining increasing attention, as reflected by Han et al., who mapped the rapidly growing field of cognitive impairment in type 2 diabetes and identified Alzheimer’s disease, insulin resistance, oxidative stress, neuroimaging, gut microbiota, and molecular mechanisms as major research themes. Viewed together, these studies suggest that diabetic complications arise from overlapping pathways involving metabolic stress, vascular injury, inflammation, hormonal dysregulation, and tissue-specific susceptibility.

Another important contribution of this collection is its emphasis on population heterogeneity across the life course. Xiang et al. showed that postmenopausal women with young-onset type 2 diabetes had markedly higher mortality risk than premenopausal women, indicating that menopausal status should be incorporated into risk stratification for women with young-onset type 2 diabetes. Liu et al. demonstrated that recurrent gestational diabetes was independently associated with hypertensive disorders in a subsequent pregnancy, particularly among women with additional maternal risk factors. Extending this life-course perspective to pediatric diabetes, Al-Abdulrazzaq et al.. found that nearly one-third of children newly diagnosed with type 1 diabetes in Kuwait were overweight or obese, and that excess weight was associated with older age, male sex, fewer diabetic ketoacidosis cases, higher C-peptide levels, and lower odds of celiac autoimmunity. These findings highlight the need for phenotype-specific diabetes care, in which reproductive stage, menopausal status, childhood obesity, residual beta-cell function, and cardiometabolic background are integrated into screening and follow-up strategies.

This Research Topic also broadens the clinical view of diabetes by addressing less traditional associations and diagnostic challenges. Song et al. reported an association between goiter and type 2 diabetes, suggesting that thyroid morphology may be linked to metabolic dysregulation, although longitudinal studies are needed to clarify causality. Tang et al. synthesized cohort evidence on diabetes and multiple sclerosis, finding that diabetes, especially type 2 diabetes, was associated with increased subsequent multiple sclerosis risk, whereas multiple sclerosis was not significantly associated with later diabetes risk. Yan et al. presented a case of variegate porphyria in an older woman with long-standing type 2 diabetes whose abdominal pain and neuropsychiatric symptoms had initially been attributed to diabetic neuropathy. These studies remind clinicians that symptoms in patients with diabetes should not always be interpreted through the lens of common diabetic complications; endocrine, immune-mediated, neurological, and rare metabolic disorders may coexist and require diagnostic vigilance.

Overall, this Research Topic shows that diabetes is a heterogeneous, multisystem disease and highlights the need to move beyond glucose-centered assessment. Across these studies, diverse clinical, digital, imaging, and omics-based indicators are integrated to support earlier detection and more individualized risk stratification. Future research should validate these tools in large, prospective, and diverse cohorts and translate them into practical clinical pathways for earlier prevention, more precise complication screening, and phenotype-based diabetes management.
